# Deep Learning Approach for Pitting Corrosion Detection in Gas Pipelines

**DOI:** 10.3390/s24113563

**Published:** 2024-05-31

**Authors:** Ivan Malashin, Vadim Tynchenko, Vladimir Nelyub, Aleksei Borodulin, Andrei Gantimurov, Nikolay V. Krysko, Nikita A. Shchipakov, Denis M. Kozlov, Andrey G. Kusyy, Dmitry Martysyuk, Andrey Galinovsky

**Affiliations:** 1 Artificial Intelligence Technology Scientific and Education Center, Department of Welding, Diagnostics and Special Robotics, Bauman Moscow State Technical University, 105005 Moscow, Russia; 2 Scientific Department, Far Eastern Federal University, 690922 Vladivostok, Russia

**Keywords:** pitting corrosion, deep neural network, gas pipelines

## Abstract

The paper introduces a computer vision methodology for detecting pitting corrosion in gas pipelines. To achieve this, a dataset comprising 576,000 images of pipelines with and without pitting corrosion was curated. A custom-designed and optimized convolutional neural network (CNN) was employed for binary classification, distinguishing between corroded and non-corroded images. This CNN architecture, despite having relatively few parameters compared to existing CNN classifiers, achieved a notably high classification accuracy of 98.44%. The proposed CNN outperformed many contemporary classifiers in its efficacy. By leveraging deep learning, this approach effectively eliminates the need for manual inspection of pipelines for pitting corrosion, thus streamlining what was previously a time-consuming and cost-ineffective process.

## 1. Introduction

In the realm of gas industry, pipelines assume an essential role in the transportation of raw materials. However, corrosion poses a significant threat to their reliability and safety. Corrosion damage can lead to structural defects, potentially resulting in gas leaks [1], accidents [2], and even emergencies. Moreover, the corrosion process is exacerbated by the operational conditions of pipelines, which are subject to aggressive environments and physical stresses. In light of this, effective methods for detecting and monitoring corrosion in gas pipelines are essential for maintaining their functionality and safety. This article explores a deep learning (DNN) approach specifically targeted at detecting pitting corrosion in gas pipelines, focusing on the identification of deep-seated depressions or holes in images of surfaces rather than overall rust levels in images. It examines the potential application of this method in enhancing the reliability and safety of gas transportation systems.

**Figure 1:**
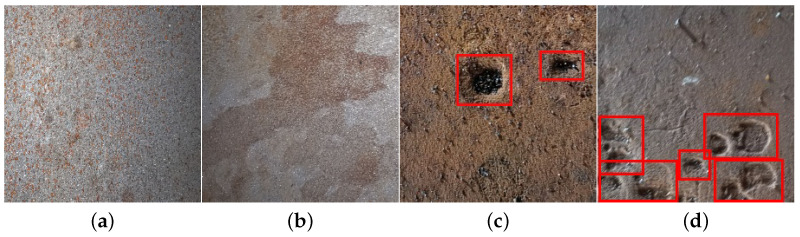
Examples of gas pipeline surfaces: (**a**,**b**) without and (**c**,**d**) with pitting corrosion.

Benign gas pipeline surface alterations refer to minor, non-corrosive irregularities [3,4] or imperfections that may occur naturally or as a result of external factors, such as handling or environmental exposure (Figure 1a,b). These alterations typically do not pose a threat [5] to the integrity or safety of the pipeline and may include surface discoloration [6], scratches [7], or minor dents [8]. On the other hand, surfaces exhibiting pitting corrosion display localized, deeper depressions [9] (Figure 1c,d) or cavities caused by chemical [10] or electrochemical [11] reactions, potentially leading to structural weakening and leaks if left untreated. Distinguishing between benign surface alterations and pitting corrosion is crucial for accurately assessing the condition of pipeline surfaces and for prioritizing maintenance and repair efforts to ensure the continued safe operation of the infrastructure.

One of the main challenges in classifying pitting corrosion, as opposed to rust levels, is the need for precise identification of deep-seated depressions [12] or holes [13] in the pipeline surface. Pitting corrosion can manifest as small but deep defects, which can be difficult to distinguish from ordinary surface irregularities or roughness. This requires image processing algorithms to be exceptionally sensitive [14] and capable of detecting even the slightest signs of pitting corrosion amid other types of defects or background noise. Additionally, minimizing false positives and ensuring high accuracy and reliability in detecting pitting corrosion are essential for the safety and reliability of pipeline systems.

The proliferation of gas pipelines, both existing and under construction, is a significant aspect of modern infrastructure development. The works in [15,16] highlighted surface corrosion as the predominant operational issue arising from external environmental factors. Such defects typically manifest as either planar, such as stress-corrosion cracks, or volumetric, notably local pitting corrosion [17]. Various non-destructive testing (NDT) methodologies are employed in the industry to identify these defects automatically. Common techniques encompass visual inspection [18], magnetic flux leakage [19], eddy-current [20], and ultrasonic methods [21]. However, a singular NDT method often fails to gather sufficient data for accurate defect classification, necessitating data fusion of multiple NDT modalities [22]. Despite advancements, these NDT methods may yield false positives, where large pitting corrosion may be erroneously identified as areas lacking transducer coupling. While pitting corrosion is visually discernible, manual inspection of pipeline surfaces via television cameras [23] proves to be data-intensive and laborious. Thus, integrating computer vision models into television inspection systems becomes imperative for efficient classification of corrosion types.

Numerous efforts have emerged in recent years focusing on external corrosion detection through television camera inspections coupled with computer vision processing. Traditional computer vision algorithms, machine learning techniques, and deep learning architectures have been employed for this purpose [24,25]. Notably, convolutional neural networks (CNNs) have gained prominence in image classification tasks [26,27]. CNNs leverage convolutions as a key mathematical operation, facilitating pixel data processing for image recognition and analysis [28]. Their efficacy spans various industries, including corrosion detection, where CNNs have been integrated into image processing workflows [29]. Recent studies have evaluated state-of-the-art CNN architectures such as ZF Net [30] and VGG16 [31] for corrosion detection, with sliding window approaches proving effective for inference [32]. DNN methods have also been compared to traditional OpenCV techniques for metal corrosion detection, demonstrating superior performance in real-world scenarios [33]. Additionally, CNN-based models have outperformed other supervised methods in corrosion grade identification, achieving high accuracy rates [34]. Deep learning approaches have been extended to corrosion segmentation tasks, with models such as FCN, U-Net, and Mask R-CNN exhibiting promising results [35].

Ossai [36] employed various machine learning techniques, including principal component analysis (PCA) [37], particle swarm optimization (PSO) [38], feed-forward artificial neural network (FFANN) [39], gradient boosting machine [40] (GBM), random forest (RF), and deep neural network (DNN) algorithms, to assess the corrosion defect depth growth in aged pipelines. By adjusting FFANN hyperparameters with PSO and utilizing PCA to transform pipeline operating variables, different ML models were developed and tested for X52-grade pipelines. Comparative analysis revealed that ML modeling with PCA-transformed data yielded a significantly higher accuracy (3.52 to 5.32 times) compared to models without PCA transformation.

To automate pipeline inspection and prioritize maintenance, a machine vision-based methodology was proposed by Bondada et al. [41]. This approach identifies and quantifies corrosion damage, aiding in the effective management of pipeline integrity and remedial measures.

Transmission pipelines, vital in the oil and gas industry, face various risks, including corrosion, with offshore pipelines particularly susceptible to pitting corrosion. Akhlagh et al. [42] investigated the efficacy of DL models, specifically generalization and generalization–memorization models, in predicting the maximum depth of pitting corrosion in these pipelines. Trained on diverse soil characteristics and pipe coating types, the deep neural networks achieved a mean squared error of 0.0055 on training data and 0.0037 on test data.

Chen et al. [43] utilized an artificial neural network (ANN) to predict the residual strength of corroded natural gas pipelines. Overcoming challenges like limited training data and overfitting, innovative techniques such as ReLU activation and dropout methods were employed. THe results showed that the multilayer perceptron (MLP) with dropout method outperformed simpler feedforward neural network (FFNN) structures and FFNN optimized by PSO, especially in scenarios with limited sample data.

Shaik et al. [44] developed an intelligent model to predict crude oil pipeline conditions based on factors like metal loss anomalies, wall thickness, weld anomalies, and pressure flow. Using a feed-forward back propagation network (FFBPN) trained with historical inspection data, the model achieved a high accuracy. Validation against other models confirmed its robustness, outperforming previous approaches.

This paper contributes to the field by developing a CNN model specifically tailored for identifying corrosion on gas pipeline external surfaces, focusing on classifying images with and without pitting corrosion. Notably, surface rust and other color variations are considered acceptable and are categorized as non-corrosion instances. The proposed model aims to distinguish images exhibiting pitting corrosion, indicative of real metal loss, from those with benign surface alterations. To the best of our knowledge, existing models primarily focus on detecting various grades of rust and other forms of damage [30,45,46], highlighting a gap in the literature. Thus, the objective of this work was to curate relevant data, develop an appropriate model, train it, and rigorously evaluate its performance.

## 2. Materials and Methods

### 2.1. Dataset

**Figure 2:**
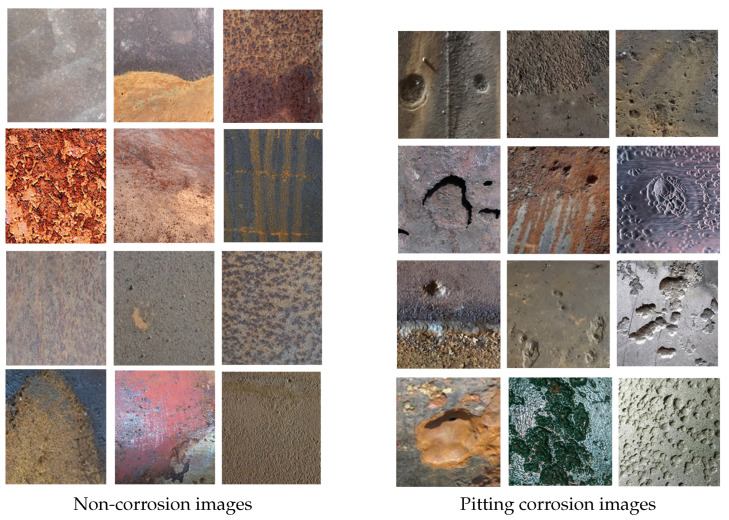
Dataset image examples.

The dataset serves as a cornerstone in the CNN training regimen. Acquiring a sufficiently extensive dataset of pipeline surfaces afflicted with pitting corrosion poses a formidable challenge, compounded by the absence of publicly available datasets tailored for pitting corrosion and non-corrosion image classification. Our team conducted comprehensive site visits to various oil and gas facilities, to execute NDT procedures and capture photographs of surface flaws from diverse perspectives. These images were subsequently standardized to dimensions of 224 × 224 pixels. Additionally, our dataset was enriched with frames extracted from pipeline television inspection videos, each resized to match the aforementioned dimensions. The finalized dataset consisted of 576,000 images, with 427,000 depicting non-corrosion instances and 149,000 portraying pitting corrosion. Examples of images from both categories are depicted in Figure 2.

Throughout model development, the dataset underwent partitioning into training, validation, and test sets via randomized image shuffling, preserving a balanced distribution of image classes across all partitions. Table 1 presents a comprehensive breakdown of the image distribution within each class across the aforementioned partitions.

Table 1 indicates a notable class imbalance within the dataset. Specifically, the number of non-corrosion images surpasses that of pitting corrosion images. This imbalance introduced additional complexity into the classification task, necessitating careful consideration during model training and evaluation.

Unlike the training dataset, which is utilized for parameter optimization, a validation dataset remains untouched during training and serves as an independent measure of a model’s generalization capability. This ensures that the model’s performance is evaluated on unseen data, thereby providing a reliable estimate of its effectiveness in real-world scenarios.

### 2.2. Model Synthesis

CNNs have emerged as powerful tools for image data analysis, dominating key tasks in image classification, object detection, and computer vision [47]. They leverage convolution processes within their architecture, allowing the efficient feature extraction that is essential for precise classification. In the realm of nNDT, CNN utilization enables the automation of defect detection on various surfaces, including metallic structures and pipelines within industrial settings. In the context of gas pipelines, where even minor defects can have serious consequences, CNNs are capable of analyzing large volumes of image data, considering factors such as lighting and texture, thereby ensuring a high accuracy in defect classification and localization.

To assess the efficacy of various CNN architectures, including AlexNet [48], ZFNet [49], VGGNet [50], Inception [51], ResNet [52], and Xception [53], we attempted to train these networks from scratch using our dataset. Feature extraction or fine-tuning of pre-trained models on the ImageNet dataset was not recommended, due to significant dissimilarities between our images and those in the ImageNet classes [54,55]. This decision was based on the significant differences between our images and those in the ImageNet dataset, which may struggle to distinguish pitting corrosion from typical rust [56,57], given its broad categories. Consequently, relying on ImageNet could have led to inaccurate results in detecting pitting corrosion, compromising the effectiveness of our classification system.

**Figure 3:**
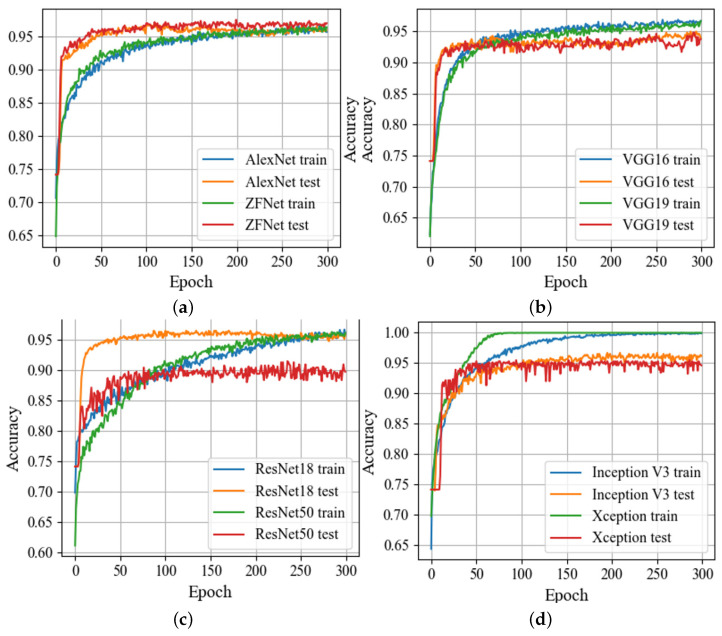
Accuracy of the given CNN architectures on the training and validation sets: (**a**) AlexNet and ZFNet; (**b**) VGG16 and VGG19; (**c**) ResNet18 and ResNet50; (**d**) Inception V3 and Xception.

Training was performed using the RMSprop optimization algorithm, with a learning rate of $1\times 10^{-5}$, incorporating $L_{2}$ regularization with a weight decay parameter of $1\times 10^{-4}$, and dropout with a probability of 0.5 for fully connected layers to prevent overfitting. Given the class imbalance in our dataset, the loss function included class weights of 1 for non-corrosion images and 2.5 for pitting corrosion images, along with label smoothing (parameter = 0.3). The CNN training process utilized a GPU Nvidia RTX 3090 with 24 GB memory and a batch size of 64 (Nvidia, Santa Clara, CA, USA). Implementation was carried out using Python 3.9 with TensorFlow 2.5.0 (Google, Mountain View, CA, USA) and OpenCV 4.5.1 (Intel, Santa Clara, CA, USA) libraries. The accuracy plots across each epoch, essential for evaluating CNN architectures, are depicted in Figure 3.

Performance evaluation on the test set involved utilizing multiple metrics, including the confusion matrix, accuracy, F1 score, area under receiver operating characteristic curve (ROC AUC), and area under precision–recall curve (P–R AUC).

The accuracy was calculated as the number of correctly classified examples divided by the total number of examples, expressed by Equation (1):

(1) $$\mathrm{Accuracy}=\frac{\mathrm{TP}\;+\;\mathrm{TN}}{\mathrm{TP}\;+\;\mathrm{TN}\;+\;\mathrm{FP}\;+\;\mathrm{FN}}$$

The F1 score is the harmonic mean of precision and recall. Precision measures the proportion of positive identifications that were actually correct, indicating fewer false positives. Recall assesses the classifier’s ability to identify all relevant instances, signifying fewer false negatives. Precision, recall, and the F1 Score were computed as shown in Equations (2), (3) and (4) respectively:

(2) $$\begin{array}{cc} \mathrm{Precision} & =\frac{\mathrm{TP}}{\mathrm{TP}\;+\;\mathrm{FP}} \end{array}$$

(3) $$\begin{array}{cc} \mathrm{Recall} & =\frac{\mathrm{TP}}{\mathrm{TP}\;+\;\mathrm{FN}} \end{array}$$

(4) $$\begin{array}{cc} F1\;\mathrm{Score} & =\frac{2\times \mathrm{Recall}\times \mathrm{Precision}}{\mathrm{Recall}+\mathrm{Precision}} \end{array}$$

The ROC curve plots the true positive rate (TPR) against the false positive rate (FPR) at various classifier threshold values. The area under the ROC curve (ROC AUC) indicates the classifier’s ability to discriminate between positive and negative classes across all possible thresholds. TPR and FPR are computed as follows:

(5) $$\begin{array}{cc} \mathrm{TPR}\;\left(\mathrm{Recall}\right) & =\frac{\mathrm{TP}}{\mathrm{TP}\;+\;\mathrm{FN}} \end{array}$$

(6) $$\begin{array}{cc} \mathrm{FPR} & =\frac{\mathrm{FP}}{\mathrm{FP}\;+\;\mathrm{TN}} \end{array}$$

Similarly, the precision–recall (P–R) curve assesses binary classification model performance, particularly in situations with imbalanced classes. The area under the P-R curve (P-R AUC) serves as a performance metric, with higher values indicating a better classifier performance.

Table 2 presents the accuracy, F1 Score, ROC AUC, and P-R AUC metrics. The F1 score was computed at the best threshold determined from the P-R curve.

**Figure 4:**
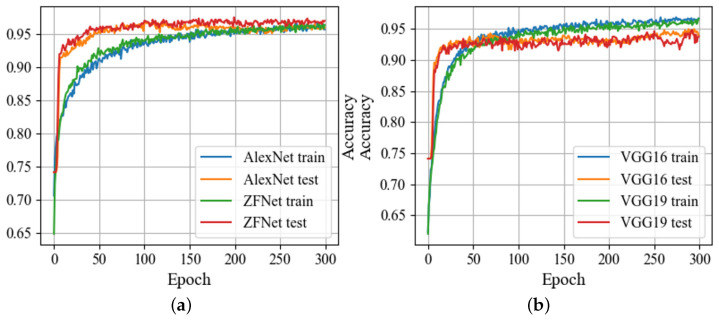
ROC (**a**) and P–R (**b**) curves of the provided architectures.

ROC curves and precision–recall (P–R) curves for all the provided CNN architectures are illustrated in Figure 4. Analyzing the results from Table 2 and Figure 3 and Figure 4, it is evident that the ZFNet architecture exhibited the best performance metrics. Deep, large networks such as VGG, ResNet, Inception, and Xception showed slight signs of overfitting on this dataset, likely due to its relatively small size compared to the ImageNet dataset. Despite this, the ZFNet architecture, a modified version of AlexNet, achieved an accuracy of 96.7% and an F1 Score of 95.3%. However, it should be noted that this network had a large number of trainable parameters. Therefore, the objective of this study was to develop a custom CNN using convolutional and fully connected layers with fewer trainable parameters, while maintaining high accuracy and other performance metrics on the test set.

Understanding the growth curves of accuracy over epochs (Figure 3) is important in assessing the performance and convergence of DNN models, by providing insights into how quickly and steadily a model learns from the training data, as well as its ability to generalize to unseen data. By analyzing these curves, it is possible to identify potential issues such as overfitting [58] or underfitting [59] and make informed decisions about the model architecture, optimization techniques, and training parameters. Comparison of accuracy across all models [60] (Table 2) allows pinpointing the most promising approaches and focusing further efforts on refining and optimizing those models for practical deployment.

## 3. Results

### 3.1. Custom Model Design and Development

The original ZFNet architecture includes a flattened layer and two fully connected layers, totaling 4096 parameters. However, fully connected layers are prone to overfitting. To address this issue, our proposed Custom CNN incorporates a global average pooling (GAP) layer [61] to reduce the number of parameters and mitigate overfitting. Additionally, the Custom CNN features a reduced number of kernels: 64 in the first two convolutional layers and 128 kernels in the subsequent convolutional layers. Through manual testing of various modifications, this configuration demonstrated optimal performance. The complete structure of the Custom CNN is outlined in Table 3.

Determining the optimal CNN architecture for this task required careful tuning of the hyper-parameters. However, conventional methods such as grid search and random search were not suitable, due to the computational expense and high iteration count involved in training complex architectures. Instead, Bayesian optimization [62] was employed in this study.

Bayesian optimization operates under the assumption of a predefined number of samples provided by the function *f*, representing various hyper-parameter combinations and their corresponding performances. Denoted as $D_{t}=\left\{\left(x_{1},f\left(x_{1}\right)\right),\left(x_{2},f\left(x_{2}\right)\right),\ldots ,\left(x_{t},f\left(x_{t}\right)\right)\right\}$, where *t* is the number of samples, and each $x_{i}$ corresponds to a specific hyper-parameter configuration. The method assumes a prior distribution P(f), leading to a posterior distribution given by

$$P\left(f|D_{t}\right)\propto P\left(D_{t}|f\right)P\left(f\right)$$

This posterior distribution aids in making informed estimates of hyper-parameter configurations based on observed performance [62]. In practice, the objective function *f* is evaluated using a surrogate function, and the next evaluation point at t+1 is determined using an acquisition function. The acquisition function balances exploration and exploitation, seeking regions where the objective function is uncertain, while exploiting areas with minimal values of *f*. Common surrogate functions include Gaussian process (GP), sequential model-based algorithm configuration (SMAC) using random forest, and tree Parzen estimators (TPE) [63].

To initiate Bayesian hyper-parameter optimization, the hyper-parameter space needs to be defined. The search space involves varying the number of kernels and kernel sizes for the convolutional layers. The hyper-parameter space for Bayesian optimization is presented in Table 4.

**Figure 5:**
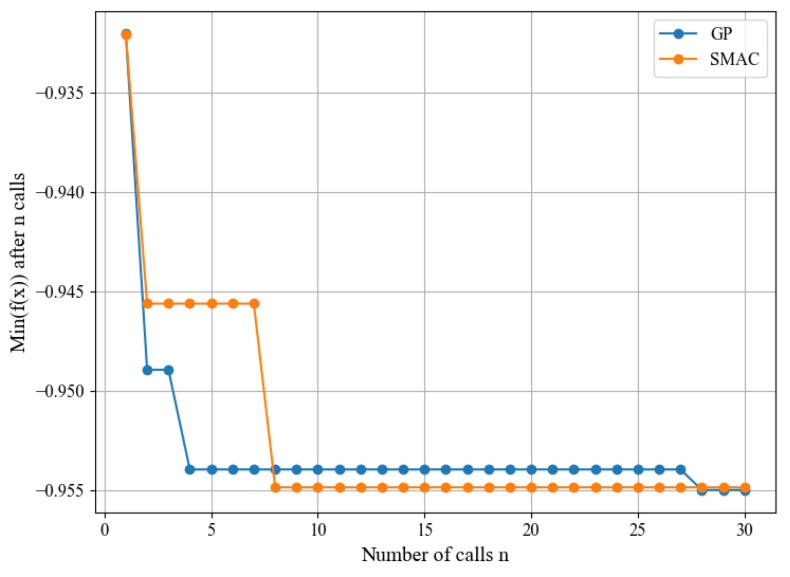
GP and SMAC convergence plots.

The initial parameters for the search were based on the Custom CNN architecture outlined in Table 3. Bayesian optimization was conducted using GP and SMAC with 30 trials. The optimization process was implemented using the Skopt library. Figure 5 illustrates the convergence plots for GP and SMAC, demonstrating the convergence of both algorithms. The objective function was minimized to optimize for the F1 score, due to the class imbalance in the dataset. The best architectures obtained using GP and SMAC are presented in Table 5 and Table 6 along with its performance testing compared to ZFNet (Table 7).

**Figure 6:**
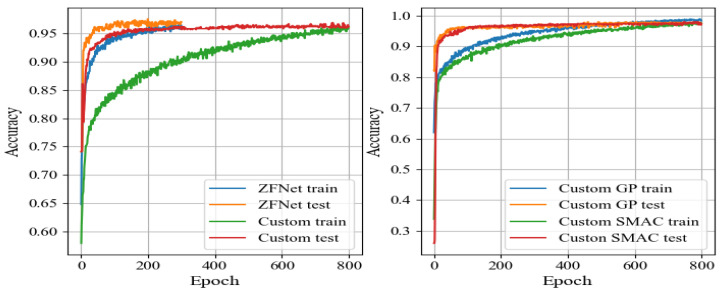
Developed CNN architecture training and validation set accuracy.

**Figure 7:**
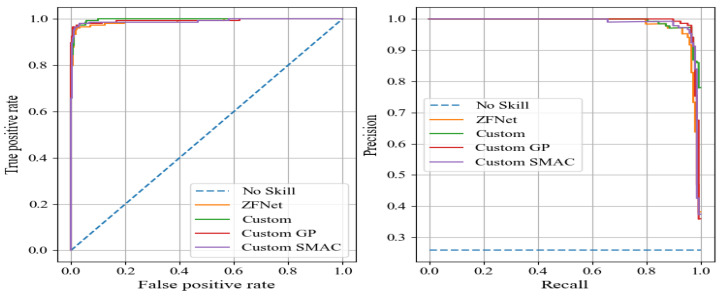
ROC and P–R curves of developed architectures and ZFNet.

**Figure 8:**
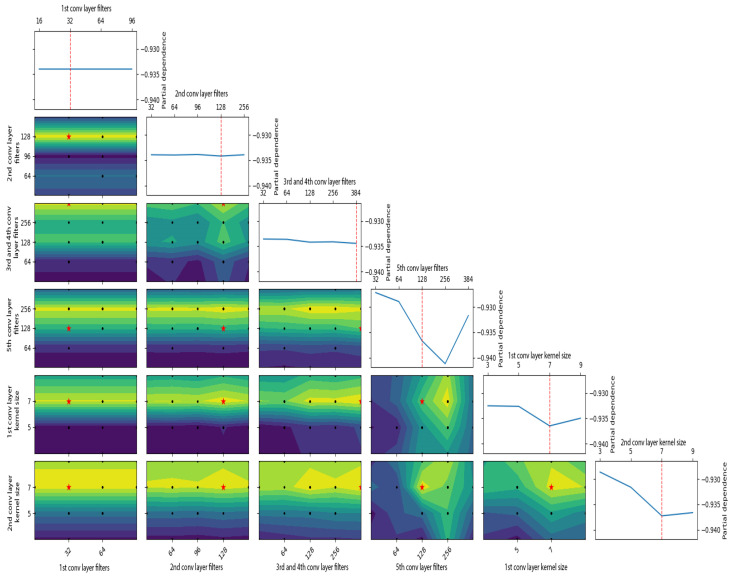
Partial dependence plot of the GP objective function.

The custom CNN architecture also boasts a reduced parameter count compared to other established architectures, as delineated in Table 8. This streamlined parameterization not only facilitates faster predictions but also minimizes memory consumption, optimizing computational efficiency and resource utilization (See Figure 6, Figure 7 and Figure 8).

### 3.2. Custom Model Performance Analysis

**Figure 9:**
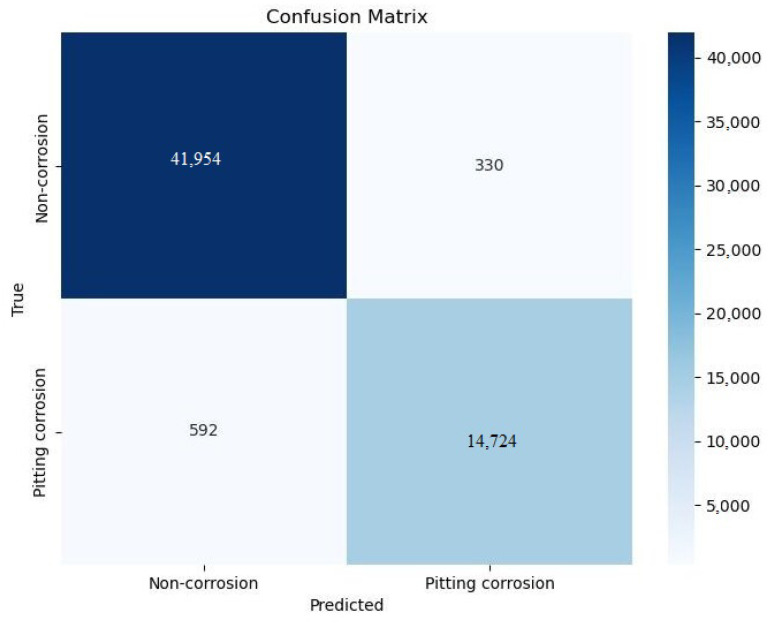
Confusion matrix of the Custom GP optimized model test set performance.

A validation set performance confusion matrix of the Custom GP optimized model is illustrated in Figure 9.

**Figure 10:**
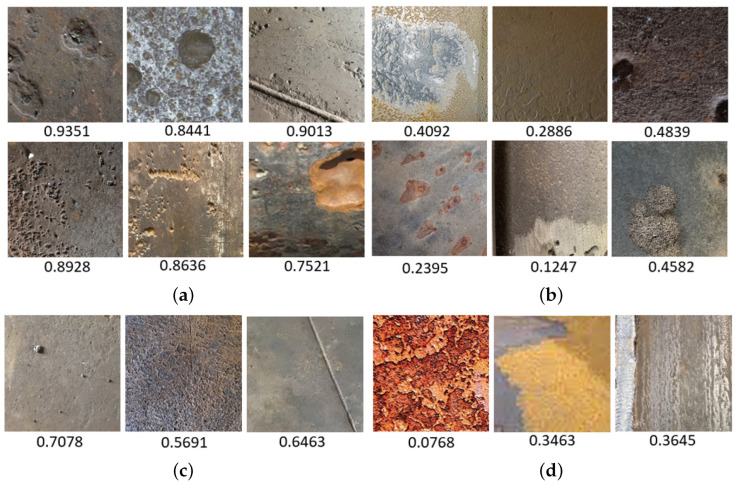
Examples of the Custom GP optimized model test set predictions with the probability values after the sigmoid activation function: (**a**) correctly predicted pitting corrosion images examples, (**b**) incorrectly predicted non-corrosion images, (**c**) incorrectly predicted non-corrosion images examples, (**d**) incorrectly predicted pitting corrosion images.

As evident from Figure 9, the model correctly predicted most parts of images (56,678 images). However, there were certain misclassifications: 592 pitting corrosion images were predicted as non-corrosion, and 330 non-corrosion images were predicted as pitting corrosion. Examples of correctly predicted images are shown in Figure 10a,c, while incorrectly predicted images are shown in Figure 10b,d.

The developed model utilizes the sigmoid activation function in the final layer, resulting in the model output as a probability (a real number between 0 and 1). If the probability is higher or equal to the threshold value (0.5), the image prediction status is labeled as “pitting corrosion”; otherwise, it is labeled as “non-corrosion”. As observed in Figure 10, pitting corrosion images consistently exhibited high probability values (more than 0.75), whereas non-corrosion images tended to have lower probabilities (not exceeding 0.4). Incorrect predictions were often associated with probabilities close to the threshold, as well as with highly cropped pit areas or extraneous elements in the image.

**Figure 11:**
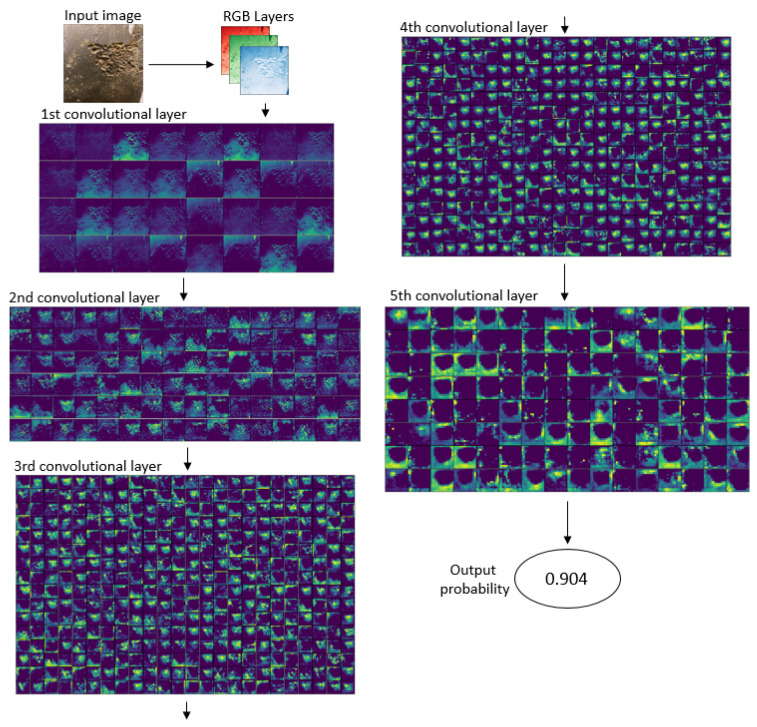
Convolutional layer output in the Custom GP optimized model.

Figure 11 displays the output of each convolutional layer in the developed model. Notably, feature maps in the initial layers capture fine details, whereas subsequent layers exhibit progressively less detailed representations. Ultimately, the model outputs the probability of pitting corrosion, enabling predictions based on a defined threshold of 0.5.

## 4. Discussion

Detection of corrosion using neural networks is a well-explored area in the scientific literature. For instance, Bastian et al. [30] presented a computer vision approach for corrosion detection in water, oil, and gas pipelines. We curated a dataset comprising over 140,000 optical pipeline images with varying corrosion levels. Employing a custom-designed CNN, we classified pipeline images based on corrosion levels. Despite its streamlined architecture, our CNN achieved a remarkable classification accuracy of 98.8%, surpassing many existing classifiers. Moreover, our proposed algorithm for corrosion localization, leveraging recursive region-based methods, had enhanced precision in identifying corroded regions within images. This deep learning methodology obviates the need for costly and disruptive manual inspections and non-vision-based evaluation techniques, significantly streamlining pipeline maintenance processes.

Unlike their approach, we focused exclusively on processing images of pitting corrosion in gas pipelines, leveraging a significantly larger dataset. This approach allowed for a more comprehensive and detailed analysis of corrosion patterns and enabled the model to learn a wider variety of features associated with corrosion, ultimately leading to more accurate and robust detection results.

Binary classification for pitting corrosion detection in images of gas pipelines presents several potential limitations. Firstly, the effectiveness of binary classification heavily relies on the quality and diversity of the training data [64]. Insufficient or biased training data may result in a poor generalization performance [65], leading to inaccurate classification outcomes, especially in real-world scenarios where environmental conditions and corrosion patterns can vary significantly.

Pitting corrosion often exhibits subtle and localized damage, making it challenging to distinguish from other types of corrosion [66] or surface irregularities. This complexity can lead to misclassifications [67] or false positives/negatives, reducing the reliability of the classification system.

Furthermore, the binary classification approach may overlook important nuances in pitting corrosion severity and progression. Pitting corrosion can occur across a spectrum of sizes and depths, each posing different levels of risk for the integrity of the pipeline [68]. Failing to account for these nuances in binary classification could result in inadequate prioritization of maintenance or repair efforts, potentially leading to safety hazards or economic losses.

Lastly, the binary classification approach may struggle to adapt to evolving corrosion patterns or conditions over time [69]. Pitting corrosion mechanisms can be influenced by various factors, such as environmental changes, pipeline material degradation, or operational parameters. A static binary classification model may lack the flexibility to adapt to such changes, necessitating periodic retraining or fine-tuning to maintain optimal performance [70].

In summary, while binary classification offers a straightforward approach to pitting corrosion detection, it is not without its limitations. Addressing these limitations requires careful consideration of factors such as training data quality, classification model robustness, and adaptability to changing corrosion conditions, to ensure reliable and effective corrosion detection in gas pipelines.

## 5. Conclusions

Oil and gas pipelines are susceptible to surface defects like pitting corrosion during their operational lifespan. While such defects are detectable through visual inspection, automating this process with television cameras can significantly enhance efficiency. However, the manual analysis of camera frames covering the entire pipeline surface is both resource-intensive and time-consuming. To address this challenge, we proposed the integration of a computer vision model within a television inspection system capable of identifying images exhibiting pitting corrosion. This study addressed the classification of 576,000 images, encompassing instances of both pitting corrosion and non-corrosion in gas pipelines. Initially, conventional CNN architectures, including AlexNet, ZFNet, VGG, ResNet, Inception, and Xception were trained on this dataset. Subsequently, a custom architecture was developed and optimized using a Gaussian process (GP) and sequential model-Based algorithm configuration (SMAC). The optimized custom architecture achieved an accuracy of 98.4%, surpassing the performance of the established CNN architectures, while exhibiting fewer parameters.

Further research directions in this domain could explore continuing to augment the dataset [71] with a more diverse range of pipeline surface images, including various types of corrosion and non-corrosion instances, which could improve the model’s robustness and generalization capabilities. Additionally, exploring novel CNN architectures or adapting [72] existing ones specifically tailored to the characteristics of pipeline surface defect detection could lead to further performance improvements. Architectures designed to handle imbalanced datasets more effectively could also be investigated. Investigating the effectiveness of transfer learning techniques [73] by fine-tuning pre-trained models on the dataset could accelerate model convergence and enhance performance, especially in scenarios with limited labeled data. Delving deeper into feature extraction methods [74] or integrating additional domain-specific features into the model pipeline could potentially enhance the model’s ability to discriminate between different types of surface defects. Exploring the fusion of information from multiple modalities [75], such as thermal imaging or ultrasonic data, alongside visual data from television inspection systems, could provide a more comprehensive understanding of surface conditions and improve the overall detection accuracy. Investigating methods for deploying the developed model in real-time television inspection systems [76], considering computational efficiency and latency constraints, would be crucial for practical deployment.

## Figures and Tables

**Table 1:** Number of images in the dataset splits.

Class	Train Set	Test Set	Validation Set	Total
Non-corrosion	341,600	42,232	43,168	427,000
Pitting corrosion	119,200	15,368	14,432	149,000
Total	460,800	57,600	57,600	576,000
Percentage	80%	10%	10%	

**Table 2:** Performance metrics of CNN architectures.

Architecture	Accuracy (%)	F1 Score (%)	ROC AUC	P-R AUC
AlexNet	95.83	92.81	0.98	0.97
ZFNet	96.70	95.30	0.98	0.98
VGG16	94.10	90.48	0.97	0.95
VGG19	94.62	92.20	0.98	0.96
ResNet18	96.18	93.52	0.99	0.98
ResNet50	91.49	84.64	0.95	0.91
Inception V3	95.66	92.05	0.98	0.97
Xception	95.31	91.84	0.98	0.97

**Table 3:** Custom CNN architecture.

No	Layer	Input	Kernel Size	Kernels	Stride	Output
1	Conv1	224 × 224 × 3	7 × 7 × 3	64	2	109 × 109 × 64
2	Pool1	109 × 109 × 64	3 × 3 × 1	-	2	54 × 54 × 64
3	Conv2	54 × 54 × 64	5 × 5 × 64	64	2	25 × 25 × 64
4	Pool2	25 × 25 × 64	3 × 3 × 1	-	2	12 × 12 × 64
5	Conv3	12 × 12 × 64	3 × 3 × 128	128	1	10 × 10 × 128
6	Conv4	10 × 10 × 128	3 × 3 × 128	128	1	8 × 8 × 128
7	Conv5	7 × 7 × 384	3 × 3 × 128	128	1	6 × 6 × 128
8	Pool3	6 × 6 × 128	3 × 3 × 1	-	2	2 × 2 × 128
9	GAP	2 × 2 × 128	-	-	-	128
10	FC	128	-	1	-	1

**Table 4:** Hyper-parameter space for Bayesian optimization.

No	Hyper-Parameter	Values
1	1st Convolution Layer Kernels	16, 32, 64, 96
2	1st Convolution Layer Kernel Sizes	3 × 3, 5 × 5, 7 × 7, 9 × 9
3	2nd Convolution Layer Kernels	32, 64, 96, 128, 256
4	2nd Convolution Layer Kernel Sizes	3 × 3, 5 × 5, 7 × 7, 9 × 9
5	3rd and 4th Convolution Layer Kernels	32, 64, 128, 256, 384
6	5th Convolution Layer Kernels	32, 64, 128, 256, 384

**Table 5:** Custom CNN architecture with GP optimization.

No	Layer	Input	Kernel Size	Kernels	Stride	Output	Parameters
1	Conv1	224 × 224 × 3	7 × 7 × 3	32	2	109 × 109 × 32	4736
2	Pool1	109 × 109 × 32	3 × 3 × 1	-	2	54 × 54 × 32	-
3	Conv2	54 × 54 × 32	7 × 7 × 32	128	2	24 × 24 × 128	200,832
4	Pool2	24 × 24 × 128	3 × 3 × 1	-	2	11 × 11 × 128	-
5	Conv3	11 × 11 × 128	3 × 3 × 128	384	1	9 × 9 × 384	442,752
6	Conv4	9 × 9 × 384	3 × 3 × 384	384	1	7 × 7 × 384	1,327,488
7	Conv5	7 × 7 × 384	3 × 3 × 384	128	1	5 × 5 × 128	442,496
8	Pool3	5 × 5 × 128	3 × 3 × 1	-	2	2 × 2 × 128	-
9	GAP	2 × 2 × 128	-	-	-	128	-
10	FC	128	-	1	-	1	129

**Table 6:** Custom CNN architecture with SMAC optimization.

No	Layer	Input	Kernel Size	Kernels	Stride	Output	Parameters
1	Conv1	224 × 224 × 3	7 × 7 × 3	16	2	109 × 109 × 16	2368
2	Pool1	109 × 109 × 16	3 × 3 × 1	-	2	54 × 54 × 16	-
3	Conv2	54 × 54 × 16	9 × 9 × 16	64	2	23 × 23 × 64	83,008
4	Pool2	23 × 23 × 64	3 × 3 × 1	-	2	11 × 11 × 64	-
5	Conv3	11 × 11 × 64	3 × 3 × 64	384	1	9 × 9 × 384	221,568
6	Conv4	9 × 9 × 384	3 × 3 × 384	384	1	7 × 7 × 384	1,327,488
7	Conv5	7 × 7 × 384	3 × 3 × 384	128	1	5 × 5 × 128	442,496
8	Pool3	5 × 5 × 128	3 × 3 × 1	-	2	2 × 2 × 128	-
9	GAP	2 × 2 × 128	-	-	-	128	-
10	FC	128	-	1	-	1	129

**Table 7:** Test set performance of the newly developed architectures compared to ZFNet.

Architecture	Accuracy (%)	F1 Score (%)	ROC AUC	P-R AUC
ZFNet	96.70	95.30	0.98	0.98
Custom	97.40	96.97	0.99	0.99
Custom GP Optimized	98.44	97.30	0.99	0.99
Custom SMAC Optimized	97.40	96.30	0.99	0.98

**Table 8:** Amount of trainable parameters in the CNN architectures.

Architecture	Amount of Trainable Parameters
ZFNet	about 30 million
VGG16	about 138 million
ResNet50	about 25.6 million
InceptionV3	about 25 million
Xception	about 22.8 million
Custom GP Optimized	about 2.4 million

## Data Availability

Data are contained within the article.
